# Distribution Patterns of Invasive Buffelgrass (*Cenchrus ciliaris*) in Mexico Estimated with Climate Niche Models under the Current and Future Climate

**DOI:** 10.3390/plants11091160

**Published:** 2022-04-26

**Authors:** Pablo Siller-Clavel, Ernesto I. Badano, Federico Villarreal-Guerrero, Jesús A. Prieto-Amparán, Alfredo Pinedo-Alvarez, Raúl Corrales-Lerma, Alan Álvarez-Holguín, Nathalie S. Hernández-Quiroz

**Affiliations:** 1 Facultad de Zootecnia y Ecología, Universidad Autónoma de Chihuahua, Periférico Francisco R. Almada km. 1, Chihuahua 31453, Mexico; pablo.siller.c@gmail.com (P.S.-C.); fvillarreal@uach.mx (F.V.-G.); jamparan@uach.mx (J.A.P.-A.); apinedo@uach.mx (A.P.-A.); rclerma@uach.mx (R.C.-L.); 2 IPICYT División de Ciencias Ambientales, Instituto Potosino de Investigación Científica y Tecnológica, Camino a la Presa San José 2055, Colonia Lomas 4a Sección, San Luis Potosí 78216, SLP, Mexico; ernesto.badano@ipicyt.edu.mx; 3 Instituto Nacional de Investigaciones Forestales, Agrícolas y Pecuarias (INIFAP), Campo Experimental La Campana, Carretera Chihuahua-Ojinaga km. 33.3, Aldama 32190, Mexico; alvarez.alan@inifap.gob.mx

**Keywords:** shared socioeconomic pathways (SSPs), regression analyses, multivariate environmental similarity surface (MESS)

## Abstract

In Mexico, buffelgrass (*Cenchrus ciliaris*) was introduced in the middle of the 20th century. Currently, buffelgrass has become an invasive species and has colonized various ecosystems in the country. In addition to its invasive capacity, climate change is a factor that has to be taken into account when considering how to effectively manage and control this species. The climatic niche models (CNM) and their projections for climate change scenarios allow for estimating the extent of biological invasions. Our study aimed to calibrate a CNM for buffelgrass in Mexico under the current climatic conditions and to project the extent of its biological invasion under climate change scenarios. For that, we used MaxEnt to generate the current CNM and to detect if climate change could cause future changes, we then evaluated the distribution patterns over the periods of 2041–2060, 2061–2080, and 2081–2100 for all the shared socioeconomic pathways (SSPs). Linear regressions were used to compare the outputs between current and future scenarios. Under the current climate, the CNM estimated that 42.2% of the continental surface of Mexico is highly suitable for buffelgrass. The regression analyses indicated no effects from climate change on the distribution of buffelgrass. Moreover, when the projected period is further in the future, and when the SSPs intensify, the surface of suitable areas for the species increases. These analyses clearly suggest Mexico is facing a biological invasion from buffelgrass, which may represent a threat to native biodiversity.

## 1. Introduction

Buffelgrass (*Cenchrus ciliaris* L., Poaceae) is a perennial species native to arid and semi-arid ecosystems of Africa. It can tolerate high temperatures that approach 50 °C [1,2]. This species was introduced to southern USA and northern Mexico for cattle foraging in the middle of the 20th century due to its resistance to drought conditions [1,3]. In Mexico, this grass spreads across semi-arid, subtropical, and tropical climates at elevations ranging from 500 to 2000 m. It is also found in marginal habitats with extreme temperatures (5.8–40.6 °C) and nutrient-poor soils, which are not usually suitable for the development of native plants [4,5]. The invasive status of this exotic grass in America, however, is a matter of debate. On the one hand, some authors postulated that buffelgrass is not an invasive plant because it neither disperses away from its introduction sites nor affects native species [2,6]. On the other hand, studies indicated buffelgrass is an aggressive invader because it quickly colonizes disturbed habitats, outcompeting native plants and altering ecosystem functioning by increasing the intensity and frequency of fires [7,8]. This affects the resilience capacity of habitats [9,10], thereby reducing the provision of ecosystems services and restricting the capacity of ecosystems to deal with a pest, among other events, which might affect the ecosystem’s resilience and make it prone to invasive species [11,12,13]. In Mexico, buffelgrass is listed in the “Agreement to determine the list of invasive exotic species for Mexico” due to the associated impacts on native biodiversity, the economy, and public health that is causes in the country [14,15]. For these reasons, and given the elevated diversity of ecosystems, which buffelgrass has colonized in Mexico, there is a need for developing tools to appropriately manage and control this species. Before the development of these tools, knowing about the potential extension (distribution) that buffelgrass could have in the country is necessary.

As climate is the primary driver of the distribution of species, modelling the climatic niches of exotic plants (i.e., the fraction of the fundamental niche that embraces those climatic factors that determine the survival of species) allows for estimating the extent that biological invasions can reach [16,17,18]. These climate niche models (hereafter, CNM) result from correlating the spatial distribution of a species (i.e., georeferenced occurrence data) with the spatial distribution of bioclimatic variables (i.e., climatic variables derived from temperature and rainfall that explain the distribution of living organisms). The geographical expression of the CNM (i.e., their projection on the physical space using geographic information systems) are maps indicating the probability of finding the target species in a given region as a function of the values these variables take across different habitats [19,20,21]. In this way, the potential distribution range of an invasive species can be estimated by determining what habitats in a region have an elevated likelihood of containing the environmental conditions for meeting its climatic niche [18].

All CNM are calibrated under the current climatic conditions because species occurrence data constitute historical records [22]. However, the management and control of invasive plants now require future projections due to the advance of climate change. The latest general circulation models of the World Climate Research Programme 6 (CMIP6 models, https://www.worldclim.org/data/cmip6/cmip6climate.html (accessed on 31 July 2021)) predict that global temperature will progressively rise during this century. This will be caused by the increased radiative forcing (i.e., the difference between in-going and outgoing energy in the Earth’s system in terms of W/m^2^), which will result from growing atmospheric concentrations of greenhouse gasses. This will also alter rainfall regimens and intensify drought in several regions [23,24]. The magnitude of these climatic changes, however, will depend on whether human societies choose to reduce carbon-dependent energy sources or continue their use. As these decisions are influenced by local and global environmental policies, the advance of carbon-free technologies, and human population growth, among other factors, four shared socioeconomic pathways (SSPs) were proposed. These SSPs describe different trends of greenhouse gases emissions, [25] and, consequently, each SSP has an associated radiative forcing level (2.6, 4.5, 7.0 and 8.5 W/m^2^) [26]. Therefore, the future distribution ranges of invasive plants can be predicted for different periods and SSPs projecting their CNM on the climatic envelopes are generated by general circulation models [27].

Considering these issues, our study was aimed (1) to calibrate a CNM for buffelgrass in Mexico to assess its invasibility under the current climatic conditions (i.e., their probability to invade; Lonsdale [28]) and (2) to evaluate the effect of climate change scenarios on the projection of the extent of this biological invasion. These projections can generate three different results, including expansions, contractions, or no changes in the distribution range of the target compared to what it has under the current climate [29,30]. As we are unable to predict the exact climatic conditions that will occur during this century, the future distribution of buffelgrass was predicted at different periods under contrasting SSPs. This information will allow for the development of adaptative control programs for this exotic plant.

## 2. Results

**Figure 1:**
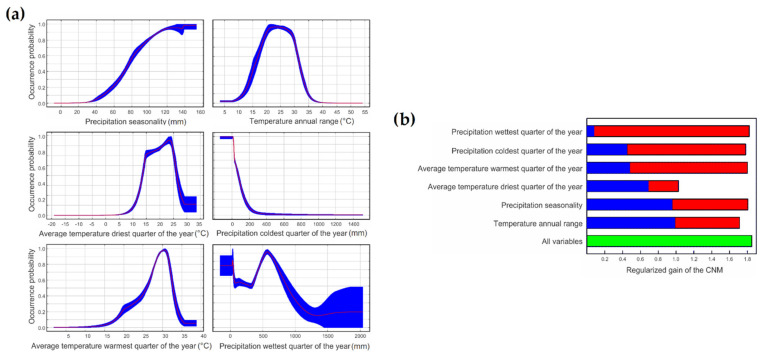
Response curves for each variable and Jackknife test. (**a**) The curves show how the predicted probability of occurrence changes as each environmental variable varies. (**b**) The Jackknife test indicates in blue the gain of the model from keeping only that variable, in red it shows the gain without the variable, and in green it shows the gain of the model with all the variables.

Most of the occurrence records used to calibrate the CNM of buffelgrass fell in Mexico (271 points = 93%), while just a few of them fell in other countries of North and Central America (17 in USA, 2 in Guatemala, and 2 in Honduras). The average AUC of the integrative CNM that resulted from the 100 bootstrap runs was 0.949 (±0.003 SE). The bioclimatic variable with the highest explanatory power in the CNM was precipitation seasonality, which accounted for 31.3% of the variance in the distribution of occurrence probabilities of buffelgrass. These values increased with higher precipitation seasonality (Figure 1a). The variable with the second highest explanatory power was the temperature annual range (explaining 23.0% of the variance; occurrence probabilities peaked around 25 °C; Figure 1). The third most important explanatory variable was the average temperature of the driest quarter of the year (explaining 19.3% variance; occurrence probabilities peaked between 15 and 25 °C; Figure 1). The fourth explanatory variable was the precipitation during the coldest quarter of the year (explaining 16.8% of variance; occurrence probabilities peaked continuously and decreased as precipitation increased; Figure 1). The bioclimatic variables with the lowest contributions to explain CNM variance were the average temperature during the warmest quarter of the year and the precipitation during the wettest quarter of the year. The former explained 5.7% of the variance and its occurrence probabilities continuously increased until temperature reached 30 °C (Figure 1), while the latter explained 4.0% of the variance and its occurrence probabilities continuously peaked around 500 mm and later decreased (Figure 1). The Jackknife test corroborated these results, indicating that precipitation seasonality and temperature annual range are the most important variables, which explain the distribution of buffelgrass. These tests also indicated that the explanatory power of the CNM is reduced if any of the bioclimatic variables used for calibration are excluded—i.e., the gain of the CNM that includes all the bioclimatic variables is lower than the gain of the models that exclude each variable (Figure 1b).

**Figure 2:**
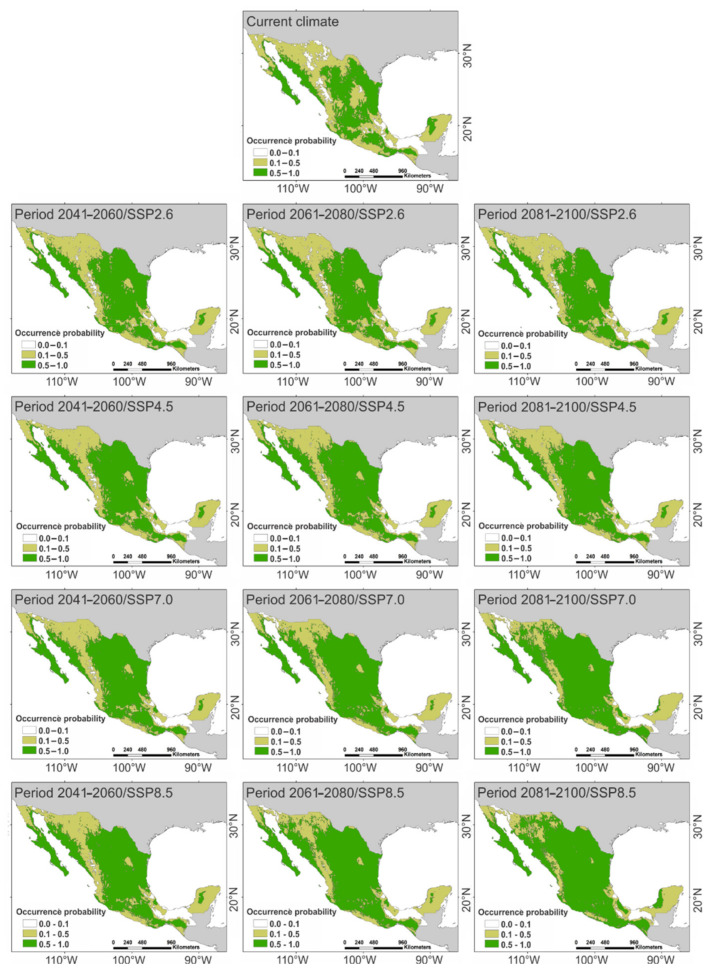
Distribution of suitable and unsuitable habitats for buffelgrass under the current and climate change scenarios. Occurrence probabilities below 0.1 are considered unsuitable, values between 0.1 to 0.5 offer moderate conditions, and above 0.5 are highly suitable habitats for the development of the species.

The geographical expression of the CNM under the current climate (Figure 2) indicated that 42.2% of the continental surface of Mexico comprises habitats that provide highly suitable climatic conditions for buffelgrass (i.e., map pixels with occurrence probabilities above 0.5 for the target species). Meanwhile, 45.6% of the country’s surface offers habitats with moderately suitable climates for the species (i.e., map pixels with occurrence probabilities between 0.1 and 0.5 for the target species). Only 12.3% of the surface of Mexico comprised climatically unsuitable habitats for the exotic species (i.e., map pixels with occurrence probabilities below 0.1 for the target species). An interactive map illustrating the current distribution of occurrence probabilities of the species estimated with the CNM is available in Supplementary Material 02 (Zenodo repository https://doi.org/10.5281/zenodo.6323654). The overlapping of this map with the climate units of Mexico indicated that warm habitats (highly warm, warm, or semi-warm climates in Table 1) with low rainfall (highly dry, dry, or semi-dry climates in Table 1) are highly susceptible to being invaded by buffelgrass. Conversely, this analysis indicated that the invasibility of habitats declines as temperature decreases and rainfall increases (temperate, semi-cold, cold climates, sub-humid, humid, or wet climates in Table 1).

**Figure 3:**
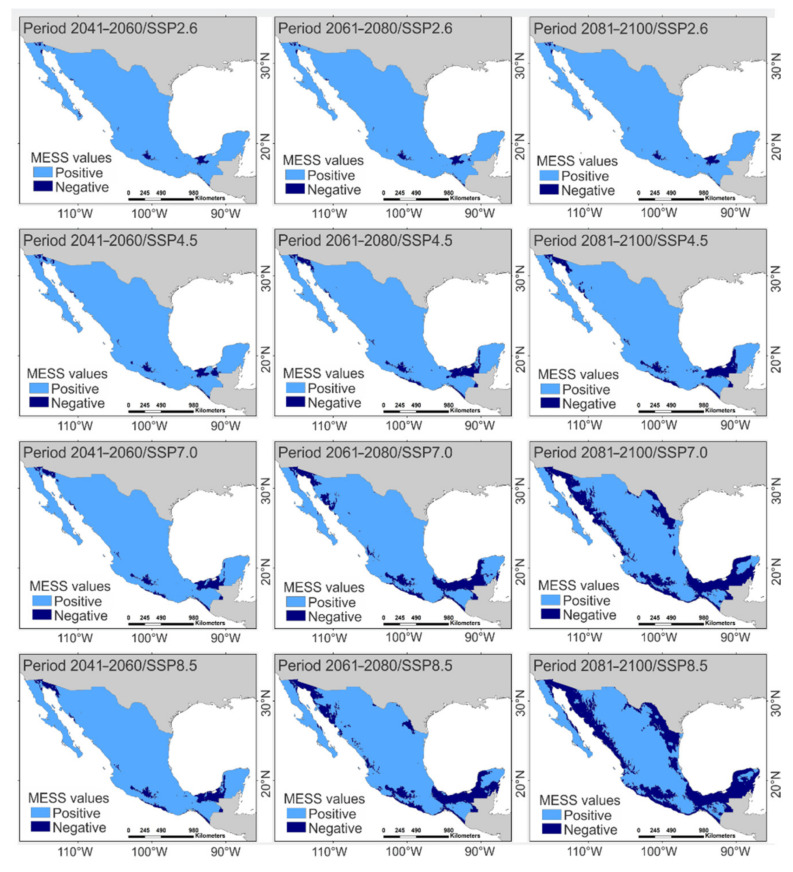
Analysis of multivariate environmental similarity surface (MESS) for each climate change scenario. Negative values indicate unsuitable habitat for the species because of climate change and positive values specify habitats that will concur with the climatic requirements of the species.

The projection of the CNM under climate change scenarios predicted that the cover of highly suitable habitats for buffelgrass (i.e., pixels with occurrence probabilities above 0.5) will rise as the projections are further in the future and radiative forcing intensifies, as compared to the cover under the current climate (Figure 2; Table 2). Conversely, the availability of moderately suitable habitats (i.e., pixels with occurrence probabilities between 0.1 and 0.5) and unsuitable habitats for the species (i.e., pixels with occurrence probabilities below 0.1) was predicted to decrease for projections further in the future and for increased radiative forcing (Figure 2; Table 2). When these predictions were superimposed on their respective MESS maps (Figure 3), the overlapping between suitable and unsuitable habitats for buffelgrass was below 10% in most climate change scenarios (Table 2). The exceptions occurred in the period of 2081–2100 with the SSPs that had higher radiative forcing (7.0 and 8.5 W/m^2^), wherein the overlapping between the predicted suitable habitats and climatically unsuitable areas for buffelgrass were above 20% (Table 2). This indicates that the CNM generates highly reliable predictions for the future distribution of buffelgrass on most climate change scenarios expected in Mexico. Meanwhile, predictions performed by the end of this century at elevated radiative forcing levels must be taken with caution, since climate change may induce extremely unfavorable environmental conditions for this exotic species. Interactive maps illustrating the future distribution of buffelgrass occurrence probabilities estimated with the CNM are available in Supplementary Material 02 (Zenodo repository https://doi.org/10.5281/zenodo.6323654).

**Figure 4:**
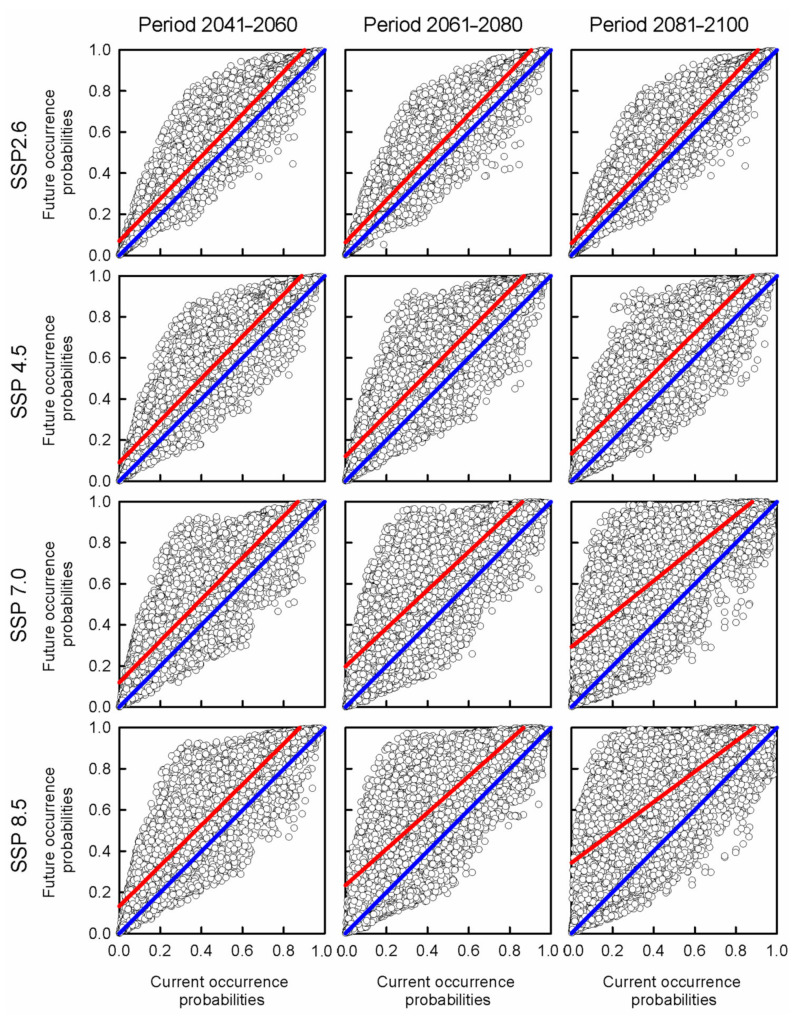
Graphs of the linear regressions analysis between current climate and climate change scenarios for the periods of 2041–2060, 2061–2080, and 2081–2100 for all the shared socioeconomic pathways. The color blue indicates the theoretical curve, and the color red shows the linear regression functions for each scenario.

The future occurrence probabilities of buffelgrass estimated with the CNM at the ten-thousand random coordinates plotted on Mexico positively related with their current occurrence probabilities for all climate change scenarios (Table 3). The intercepts and slopes of all these linear regression functions significantly differed from the theoretical values (β_0_ = 0 and β_1_ = 1). The theoretical values represented the expected values from no effects of climate change on the distribution of buffelgrass (Table 3). In all climate change scenarios, the empirical regression curves dropped above the theoretical curve (Figure 4).

## 3. Discussion

The elevated AUC of the CNM for buffelgrass, calibrated with data from North and Central America, shows the estimates of the model with elevated accuracy, thereby indicating the distribution of occurrence probabilities of this exotic species in Mexico. This has been indicated for most species’ distribution models with AUC values above 0.9 [17,18,31,32,33]. The geographical projection of the CNM suggested that warm habitats under the current climate with limited precipitation are more susceptible to become invaded by buffelgrass (i.e., they have a high invasibility) than temperate, cold, and wet habitats of this country. Our findings concur with the generalized suggestion that after its introduction in new regions, buffelgrass mainly colonizes arid and semi-arid habitats [1]. Similar results have been reported with the application of a different software to model the distribution of buffelgrass [4]. A recently developed distribution model, focusing on southern USA and northern Mexico, indicated that the invasibility of habitats increases with rising annual mean temperature and decreasing annual rainfall [34]. However, these environmental variables were not included in our model, since they were spatially autocorrelated with the bioclimatic variables used to calibrate the CNM of buffelgrass. This exclusion of annual mean temperature and annual precipitation from our model could be a consequence of considering a larger geographical extent than that used in studies focused on particular fractions of the buffelgrass distribution range. The increases or reductions in the spatial extent of CNM can change the nature of the bioclimatic variables, thereby explaining the distribution of species [35].

In our study, the bioclimatic variables with higher explanatory power on the distribution of buffelgrass in Mexico were precipitation seasonality and temperature annual range. High probabilities of occurrence were estimated for precipitations from 120 mm to 160 mm and temperatures between 15 °C and 30 °C. These results suggest that buffelgrass would prefer habitats where rainfall concentrates in a particular period of the year. Since Mexico has tropical cyclones that generate summer storms frequently [36], it is feasible to propose that the elevated explanatory power of this bioclimatic variable in the CNM occurs because the species recruits in this rainy period. In addition, these results suggest buffelgrass would mainly invade habitats where temperature widely varies across the year, which is a typical climatic feature of arid and semi-arid Mexican ecosystems [37]. These suggestions concur with other findings, which have indicated that this exotic grass can get established at habitats where annual rainfall is below 300 mm and annual temperature ranges from −5 to 45 °C [1,2,38].

The projection of the CNM on climate change scenarios suggested that the distribution range of buffelgrass will continually expand during this century. This may be due to the increasing availability of suitable habitats for the species across Mexico, with larger expansions of the distribution range at a higher radiative forcing. In other words, the invasibility of habitats is predicted to increase with larger climatic changes. In contrast, de Albuquerque [27] reported that buffelgrass will decrease in the tropical zones of the Sonoran Desert and the lowest part of the Colorado River. Our predictions are supported by the results of the regression analyses conducted between future and current occurrence probabilities, which were estimated with the CNM. Such results indicated that the occurrence probabilities of this exotic plant will intensify across habitats of Mexico in all climate change scenarios (i.e., empirical regression curves drop above the theoretical curve with β_0_ = 0 and β_1_ = 1). Thus, the ecological niche of buffelgrass seems to coincide with the environmental conditions that predominate in warm drylands [1]. The predicted expansion of its distribution range is supported by the application of regional circulation models in Mexico. This indicates increases in temperature and decreases in rainfall due to climate change, which will increase aridity across this country [39,40].

An important caveat about the current and future invasibility of habitats reported in this study is that the CNM considers that buffelgrass can colonize all climatically suitable habitats available in Mexico. This is a common assumption for models estimating the distribution of species relying on climatic variables only [27,41,42]. Thus, the geographical expression of our CNM illustrates the potential distribution range that buffelgrass can reach in the absence of natural enemies and dispersal limitations. However, as these factors might also influence the establishment of species [43,44,45], they must be discussed to assess whether the current and future distribution ranges of buffelgrass estimated with the CNM are reliable.

In Mexico, buffelgrass is promoted as cattle forage, and in production fields these plants are commonly attacked by the pathogenic fungus *Magnaporthe oryzae* (Hebert) Barr (Magnaporthaceae), thereby reducing seed harvesting [46]. Given the extensive meadows this species has developed in human-disturbed sites of northern Mexico [47], it is feasible to propose that parasites and predators have negligible effects on naturally established populations of buffelgrass. It is important to note that buffelgrass seeds are dispersed by wind and water run-off, which are common dispersal mechanisms in successful invasive plants [48]. In addition, buffelgrass seeds are often dispersed in mud attached to vehicles, while they can also adhere to cattle fur and reach remote areas that are inaccessible to vehicles [49]. This variety of highly efficient dispersal mechanisms suggests that buffelgrass seeds can reach habitats located far away from parental populations, making it unfeasible to postulate that the species is affected by dispersal limitations. These lines of evidence suggest that both the current and future distribution ranges that were estimated with the CNM of buffelgrass in Mexico are reliable enough to be used for developing regional management and control programs for this exotic species.

## 4. Materials and Methods

Although our study focused on continental Mexico only, the occurrence data required for calibrating the CNM of buffelgrass were gathered across North and Central America to cover an extensive range of environmental conditions where the species is present, as this improves the predictive accuracy of the model. This information was obtained from the Global Biodiversity Information Facility (https://doi.org/10.15468/dl.acn5rp (accessed on 9 December 2019)). This online platform compiles the largest collection of corroborated plant records, even those located in regions where species are exotic [50]. We visualized the occurrence data in QGIS 3.12.0 (available at https://qgis.org (accessed on 15 August 2021)) and removed those points located within cities and towns to avoid presences due to human interventions (e.g., unintentional water supply) that might locate the species at sites where climate is not necessarily favorable for its development [17,18]. Climate data associated with these occurrence points were obtained from the WorldClim geodatabases (available at http://worldclim.org (accessed on 31 July 2021)), which interpolate temperature and rainfall information gathered between 1970 and 2000 to estimate the current worldwide values of 19 bioclimatic variables commonly used to explain species distribution [51]. WorldClim also downscales the predictions of CMIP6 general circulation models to estimate the values of these bioclimatic variables in climate change scenarios, but since the finest resolution at which these data are provided is 2.5 arc-minutes (~21 km^2^/pixel), the buffelgrass CNM was calibrated at this spatial scale.

Once the spatial scale of the CNM was defined, we performed a new depuration of buffelgrass presences to avoid including duplicated information within the same spatial unit, as this may lead to model overfitting [52,53]. For this, we drew a circular buffer of 2.5-km radius around each occurrence point and retained a single, randomly selected record when two or more buffers overlapped. This resulted in 292 occurrence points for the modeling procedure. Given CNM may overpredict species occurrence probabilities when redundant environmental variables are included in their calibration [22,54], we conducted Spearman rank-order tests among all pairwise combinations of bioclimatic variables (i.e., bioclimatic data associated with occurrence points). We ran these analyses in R 4.0 software (available at http://cran.r-project.org (accessed on 15 August 2021)) and, when correlation coefficients above 0.60 were found, we only retained the variable that had the highest number of correlations with others. Besides minimizing environmental redundancy, this allows for an explanation of the distribution of species with relevant bioclimatic variables only [55]. With these analyses we selected six bioclimatic variables, including temperature annual range, average temperature of the driest quarter of the year, average temperature of the warmest quarter of the year, precipitation seasonality, precipitation of the wettest quarter of the year, and precipitation of the coldest quarter of the year. The dataset with the occurrence points of buffelgrass and their associated values of bioclimatic variables, as well as the results of the correlation tests used to select bioclimatic variables for CNM, are available in Supplementary Material 01 (deposited in the Zenodo repository https://doi.org/10.5281/zenodo.6323654).

We calibrated the CNM of buffelgrass with MaxEnt 3.4 software [56] because its maximum entropy algorithm performs better than other computer routines (e.g., GARP) for estimating the occurrence probabilities of species when presence-only data are available, as was our case [22,54,56]. We instructed MaxEnt to randomly select 75% of the occurrence data to calibrate the CMN (training points), while the remaining 25% of the buffelgrass data were used to validate the resulting model (test points) with receiver operating characteristic curves. These curves indicate the fraction of test points correctly classified by the CNM, while the area under the curves (AUC) estimates the accuracy of the model to estimate the occurrence probabilities of the target species as a function of the bioclimatic variables used to calibrate it. AUC varies between one (1) and zero (0), where values close to 1 indicate the CNM properly estimates the occurrence probabilities of the species in the physical space and values below 0.5 indicate that the CNM cannot differentiate among suitable and unsuitable habitats for the species [56]. To prevent potential biases due to the random selection of training points, the CNM was calibrated 100 times using the bootstrap resampling procedure of MaxEnt. After that, we integrated the 100 outputs in a single CNM and averaged their AUC to assess the accuracy of the integrative model [20]. Furthermore, we used the jackknife test of MaxEnt to identify what bioclimatic variables have important individual effects on the distribution of buffelgrass. This procedure generates a single-variable model for each environmental variable used to calibrate the CNM to identify which of them make larger contributions to the model gain (goodness of fit of the full model). Meanwhile, the Jackknife test generates models that exclude each of these variables, while including the rest of them, and compare the gain of these models against the gain of the full model. In these comparisons, an environmental variable is assumed as relevant to explain the distribution of the target species if the model that excludes it has a lower gain than the full model [57].

We plotted the CNM of buffelgrass in QGIS using an environmental background with current values for the bioclimatic variables, which were used to calibrate it, and this resulted in a map whose pixels have occurrence probabilities ranging from zero (0) to one (1). For identifying which sites have an elevated likelihood of containing the species, we reclassified the pixels of this map in probability deciles and superimposed on it the occurrence points of buffelgrass for counting the number of true presences at each decile. Since only 1.3% of buffelgrass presences dropped in pixels with occurrence probabilities below 0.1, we assumed the pixels in the lowest probability decile (0.0–0.1) constituted habitats climatically unsuitable for the species. The accumulated fraction of true presences increased to 18.9% in pixels with occurrence probabilities between 0.1 and 0.5; however, 79.8% of buffelgrass presences were concentrated in pixels with occurrence probabilities above 0.5. The number of buffelgrass occurrences that dropped at each probability decile can be consulted in Supplementary Material 01 (deposited in the Zenodo repository https://doi.org/10.5281/zenodo.6323655). Considering these values, we assumed that pixels with occurrence probabilities between 0.1 and 0.5 are habitats that offer moderately suitable climatic conditions for buffelgrass. Meanwhile, pixels with occurrence probabilities above 0.5 are highly suitable habitats for the development of the species. After that, we constrained this occurrence probability map to the continental surface of Mexico and superimposed it on a map containing the climate units of this country (available at https://www.inegi.org.mx (accessed on 9 December 2019)). According to the latter, Mexico can be split into 21 climate units (Table 1). Within each climate unit, we computed the degree of overlapping among highly suitable, moderately suitable, and unsuitable habitats to identify the most susceptible to become invaded by buffelgrass under the current climatic conditions (i.e., we assessed the invasibility of these habitats in accordance to Lonsdale [28]).

The future distribution of buffelgrass was estimated using the predictions of the Canadian Earth System Model version 5 (CanESM5) as a climatic background. For that, we chose CanESM5 because it has a higher equilibrium climate sensitivity and transient climate response than other CMIP6 models included in WorldClim [58]. As these parameters estimate the thermal sensitivity of the Earth’s system in the face of rising concentrations of greenhouse gases, the elevated values of CanESM5 make its predictions highly realistic [59,60]. Given the climate predictions from the CanESM5 cover for 20-year intervals until the end of this century (2041–2060, 2061–2080, and 2081–2100), we estimated the future distribution of buffelgrass for all of these periods considering the radiative forcing associated with the four SSP (2.6, 4.5, 7.0, and 8.5 W/m^2^). Then, we projected the CNM calibrated at each bootstrap run on all climate change scenarios (three periods × 4 SSPs = 12 climate change scenarios) and then integrated the output of these projections in a single prediction for each climate change scenario. These predictions were visualized as maps of occurrence probabilities whose pixels were classified using the aforementioned criteria. To assess the reliability of these predictions, we ran a multivariate environmental similarity surface (MESS) analysis each time the CNM was projected on a climate change scenario. The MESS analyses compare the current and future values of the bioclimatic variables used to calibrate the CNM across the target region. It differentiates among habitats that will become unsuitable for the species due to climate change (pixels with negative MESS values) and habitats that will concur with the climatic requirements of the species (pixels with positive MESS values) [27]. For each climate change scenario, we integrated the outputs of the MESS analyses that resulted from the 100 bootstrap runs in a single MESS output and expressed it as a map with pixels categorized as positive or negative. On these MESS maps, we superimposed the occurrence probabilities of buffelgrass predicted at the respective climate change scenario and calculated the percent overlapping of moderately and highly suitable habitats (i.e., pixels with occurrence probabilities above 0.1) with areas classified as unsuitable for the species in the MESS maps (i.e., areas with negative MESS values). For this procedure, we assumed that the reliability of the CNM to predict the future distribution of the exotic species increases as this percentage decreases [18].

To determine whether climate change will substantially affect the distribution of buffelgrass in Mexico, we compared the future distribution of occurrence probabilities for the species against the current distribution of occurrence probabilities. For this, we generated ten-thousand random geographic coordinates across the continental Mexico and plotted them on the current and future occurrence probabilities maps estimated with the CNM. After that, we extracted the probability values associated to each of these points and performed simple linear regression analyses using the future occurrence probabilities as response variables and the current occurrence probabilities as predictive variables. These analyses were conducted separately for each climate change scenario in R 4.0. software. In all the cases, we obtained a linear regression function of the type P*_f_* = β_0_ + β_1_ P*_c_*, where P*_f_* is the future occurrence probability of the species at a given point of the geographical space and P*_c_* is the current occurrence probability of the species at the same geographical coordinates. Meanwhile, β_0_ and β_1_ represent the intercept and slope of the regression function, respectively, which were estimated with the method of least squares [61]. This approach allows for proposing the following null hypothesis: if climate change has no effects on the distribution of buffelgrass, its current and future occurrence probabilities should be similar across the geographical space, and, therefore, the parameters of the empirical regression function should not differ from their theoretical values (β_0_ = 0 and β_1_ = 1). Alternatively, if these regression parameters differ from their theoretical values (i.e., β_0_ ≠ 0 and/or β_1_ ≠ 1), the former null hypothesis must be rejected. We used Student’s *t*-test to determine whether empirical and theoretical values of β_0_ and β_1_ statistically differ.

## 5. Conclusions

The CNM calibrated in this study indicates that the warm drylands of Mexico constitute habitats that are highly susceptible to being invaded by buffelgrass. In addition, as the advance of climate change will increase aridity across this country, the model predicts that buffelgrass populations will continually expand during this century. These analyses clearly suggest that Mexico is facing a biological invasion by buffelgrass and given the aggressive invasiveness of the species, it may constitute a threat for native biodiversity. Therefore, regional management and control programs should be implemented to prevent the expansion of buffelgrass populations. These programs must also consider that habitat invasibility will increase in the future, which could make it harder to control this biological invasion.

## Figures and Tables

**Table 1:** Results of the overlapping between the occurrence probability map of buffelgrass under the current climate and the map of the climate units of Mexico. For each climate unit, the table indicates the percent cover of habitats with highly suitable climatic conditions (occurrence probabilities above 0.5), moderately suitable climatic conditions (occurrence probabilities between 0.1 and 0.5), and unsuitable climatic conditions (occurrence probabilities below 0.1) for buffelgrass.

Climate Unit	Highly Suitable Habitats	Moderately Suitable Habitats	Unsuitable Habitats
Cold	0%	8%	92%
Semi-cold semi-dry	0%	48%	52%
Semi-cold sub-humid	0%	31%	69%
Temperate dry	30%	65%	5%
Temperate highly dry	3%	72%	26%
Temperate humid	1%	54%	45%
Temperate semi-dry	45%	53%	3%
Temperate sub-humid	37%	54%	9%
Semi-warm dry	66%	34%	0%
Semi-warm highly dry	51%	47%	2%
Semi-warm humid	10%	43%	46%
Semi-warm semi-dry	62%	38%	0%
Semi-warm sub-humid	73%	27%	0%
Warm dry	89%	11%	0%
Warm highly dry	84%	15%	2%
Warm semi-dry	94%	6%	0%
Warm sub-humid	37%	60%	2%
Warm wet	0%	14%	86%
Highly warm highly dry	100%	0%	0%
Highly warm semi-dry	85%	15%	0%
Highly warm dry	91%	9%	0%

**Table 2:** Percent cover of highly suitable habitats (pixels with occurrence probabilities above 0.5), moderately suitable habitats (pixels with occurrence probabilities between 0.1 and 0.5), and unsuitable habitats (pixels with occurrence probabilities below 0.1) for buffelgrass estimated with the CNM under the current climate and the climate change scenarios predicted by CanESM5 on 20-year intervals considering the radiative forcing associated with the different SSPs. The last column of the table shows the percent overlapping of habitats predicted as moderately and highly suitable for buffelgrass (i.e., occurrence probabilities above 0.1) with areas containing negative MESS values at each climate change scenario.

Climate Scenario		Highly Suitable Habitats	Moderately Suitable Habitats	Unsuitable Habitats	Overlapping with Negative MESS Areas
Current climate		42.2%	45.6%	12.3%	–
2041–2060	SSP2.6	SSP2.6	52.4%	39.2%	8.4%
	SSP4.5	SSP4.5	54.5%	37.6%	7.9%
	SSP7.0	SSP7.0	56.8%	35.8%	7.4%
	SSP8.5	SSP8.5	58.1%	34.6%	7.3%
2061–2080	SSP2.6	SSP2.6	52.1%	39.1%	8.7%
	SSP4.5	SSP4.5	57.2%	35.4%	7.5%
	SSP7.0	SSP7.0	62.2%	31.0%	6.9%
	SSP8.5	SSP8.5	64.2%	29.3%	6.6%
2081–2100	SSP2.6	SSP2.6	51.4%	39.8%	8.8%
	SSP4.5	SSP4.5	57.9%	34.6%	7.5%
	SSP7.0	SSP7.0	68.1%	25.9%	6.1%
	SSP8.5	SSP8.5	72.0%	22.7%	5.2%

**Table 3:** Result of the regression analyses conducted between current and future occurrence probabilities of buffelgrass across Mexico. Future occurrence probabilities were estimated by projecting the CNM on the current climate and the climate change scenarios predicted by CanESM5 on 20-year intervals considering the radiative forcing associated with the different SSPs. The table also provides the linear regression function that resulted from these analyses, where P*_f_* is the future occurrence probability of the species and P*_c_* is the current occurrence probability of the species.

Climate Scenario		Results of Regression Analyses	Empirical Linear Regression Function
2041–2060	SSP2.6	F_(1, 9998)_ = 80,688.383, *p* < 0.001, R^2^ = 0.890	$P_{f}$ = 0.069 * + 1.032 * P_c_
	SSP4.5	F_(1, 9998)_ = 59,827.316, *p* < 0.001, R^2^ = 0.857	P_f_ = 0.090 * + 1.025 * P_c_
	SSP7.0	F_(1, 9998)_ = 43,507.023, *p* < 0.001, R^2^ = 0.813	P_f_ = 0.119 * + 1.013 * P_c_
	SSP8.5	F_(1, 9998)_ = 40,987.125, *p* < 0.001, R^2^ = 0.804	P_f_ = 0.132 * + 0.988 * *P_c_*
2061–2080	SSP2.6	F_(1, 9998)_ = 80,439.742, *p* < 0.001, R^2^ = 0.889	P_f_ = 0.063 * + 1.036 * P_c_
	SSP4.5	F_(1, 9998)_ = 41,775.102, *p* < 0.001, R^2^ = 0.807	P_f_ = 0.121 * + 1.010 * P_c_
	SSP7.0	F_(1, 9998)_ = 24,058.211, *p* < 0.001, R^2^ = 0.706	P_f_ = 0.198 * + 0.931 * P_c_
	SSP8.5	F_(1, 9998)_ = 19,815.479, *p* < 0.001, R^2^ = 0.665	P_f_ = 0.234 * + 0.884 * P_c_
2081–2100	SSP2.6	F_(1, 9998)_ = 84,131.766, *p* < 0.001, R^2^ = 0.894	P_f_ = 0.058 * + 1.038 * P_c_
	SSP4.5	F_(1, 9998)_ = 36,641.332, *p* < 0.001, R^2^ = 0.786	P_f_ = 0.133 * + 0.981 * P_c_
	SSP7.0	F_(1, 9998)_ = 13,323.927, *p* < 0.001, R^2^ = 0.571	P_f_ = 0.293 * + 0.804 * P_c_
	SSP8.5	F_(1, 9998)_ = 10,316.182, *p* < 0.001, R^2^ = 0.508	P_f_ = 0.344 * + 0.738 * *P_c_*

* The empirical value statistically differs from the theoretical value (*t*-tests critical α = 0.05).

## Data Availability

All the data presented in this study are available in the article and in Supplementary Materials.

## Supplementary Materials

The following supporting information can be downloaded at: https://www.mdpi.com/article/10.3390/plants11091160/s1, Supplementary material 01, excel file that contains the information that supports the analyses performed in the study, Supplementary material 02, Keyhole Markup Language file with maps that can be visualized in Google Earth.
